# Effect of inulin-type fructans on appetite in patients with type 2 diabetes: a randomised controlled crossover trial

**DOI:** 10.1017/jns.2021.70

**Published:** 2021-09-10

**Authors:** Eline Birkeland, Sedegheh Gharagozlian, Kåre I. Birkeland, Oda K. S. Holm, Per M. Thorsby, Anne-Marie Aas

**Affiliations:** 1 Section of Nutrition and Dietetics, Department of Clinical Service, Division of Medicine, Oslo University Hospital, Oslo, Norway; 2 Faculty of Medicine, Institute of Clinical Medicine, University of Oslo, Oslo, Norway; 3 Department of Transplantation Medicine, Oslo University Hospital, Oslo, Norway; 4 Department of Nutrition, Faculty of Medicine, University of Oslo, Oslo, Norway; 5 Hormone Laboratory, Department of Medical Biochemistry, Oslo University Hospital, Oslo, Norway

**Keywords:** Diabetes type 2, Ghrelin, Prebiotics, PYY, Standardised mixed meal, GLP-1, glucagon-like peptide-1, ITF, inulin-type fructans, PYY, peptide YY, VAS, visual analogue scale

## Abstract

The aim of the study was to investigate the effect of prebiotic fibres on appetite-regulating hormones, subjective feeling of appetite and energy intake in subjects with type 2 diabetes. Data presented are secondary outcomes of a study investigating the effect of prebiotics on glucagon-like peptide-1 and glycaemic regulation. We conducted a randomised and placebo-controlled crossover trial to evaluate the effects of 16 g/d of inulin-type fructans or a control supplement (maltodextrin) for 6 weeks in randomised order, with a 4-week washout period in-between, on appetite in thirty-five men and women with type 2 diabetes. Data were collected at visits before and after each treatment: plasma concentration of the satiety-related peptides ghrelin and peptide YY (PYY) were assessed during a standardised mixed meal. The subjective sensation of appetite was evaluated in response to an *ad libitum* lunch by rating the visual analogue scale. Twenty-nine individuals (twelve women) were included in the analyses. Compared to control treatment, the prebiotics did not affect ghrelin (*P* =0⋅71) or the ratings of hunger (*P* = 0⋅62), satiety (*P* = 0⋅56), fullness (*P* = 0⋅73) or prospective food consumption (*P* = 0⋅98). Energy intake also did not differ between the treatments. However, the response of PYY increased significantly after the control treatment with mean (sem) 11⋅1 (4⋅3) pg/ml when compared to the prebiotics −0⋅3 (4⋅3) pg/ml (*P* = 0⋅013). We observed no effect of inulin-type fructans on appetite hormones, subjective feeling of appetite or energy intake in patients with type 2 diabetes.

## Introduction

Overweight and obesity represent a global epidemic, associated with comorbidities such as type 2 diabetes, unfavourable alterations of gut bacteria and low-grade inflammation^(1)^. Type 2 diabetes alone poses a considerable health risk, causing painful, disabling and life-threatening complications^(2,3)^. Lifestyle interventions, including weight regulation, physical activity and dietary adjustments, play a key role in the treatment of type 2 diabetes^(4)^, and weight reduction may potentially lead to remission^(5)^. Regulation of appetite is complex and not yet fully understood. It involves communication between the gut and the brain with positive and negative autonomic and hormonal feedback signals^(6)^. In recent years, the use of novel food ingredients has received increased attention in the prevention and treatment of overweight and its comorbidities. Among these are the non-digestible prebiotic fibres that are favoured and fermented by gut bacteria associated with improved health, of which inulin-type fructans (ITF) and galacto-oligosaccharides are the most studied^(7)^. The ITF are chains of three to sixty units of D-fructose, which are linked together with beta(2-1) glucosidic bonds and usually have a D-glucose unit at one end^(8)^. ITF with fructose units of less than ten are often called oligofructose or fructo-oligosaccharides, with the latter sometimes reserved for ITF synthesised from sucrose. The prebiotic fibres are fermented into short-chain fatty acids that may bind to receptors in enteroendocrine cells in the distal gut and trigger the increased secretion of gut hormones^(9,10)^. In turn, these hormones regulate appetite by affecting the brain and the gastrointestinal system. As different prebiotic fibres and various degrees of polymerisation may nourish different bacterial species in the gut, variation in impact on appetite is plausible.

Randomised controlled trials have shown desirable effects of prebiotics on appetite regulation, suppression of energy intake and weight loss^(11–15)^, but these trials have for the most part been conducted in non-diabetic populations. Studies report that the microbiota differs between patients with type 2 diabetes and healthy people^(16)^. There are also studies indicating that the response of an intervention with prebiotic fibres may depend on initial microbial^(17)^ and metabolic phenotype and that insulin-resistant subjects may respond differently from insulin-sensitive subjects^(18)^. Moreover, suppression of appetite and reduced energy intake could benefit the health of patients with type 2 diabetes in particular. The aim of this study was thus to investigate the impact of the prebiotic fibre inulin and oligofructose on changes in appetite hormones, subjective rating of appetite and energy intake in subjects with type 2 diabetes.

## Subjects and methods

This randomised, placebo-controlled and double-blind crossover trial was conducted between February 2016 and December 2017 at the Diabetes Research Laboratory of Oslo University Hospital, Aker. We chose a crossover approach because of the high inter-individual variability in microbial response to dietary interventions, thus allowing each participant to serve as its own control. The data presented in this present paper are secondary outcomes of a study investigating the effect of prebiotics on glucagon-like peptide-1 (GLP-1) and glycaemic regulation. We previously reported that despite inducing moderate changes in the composition of faecal bacteria and increasing faecal concentrations of short-chain fatty acids, the prebiotics did not positively affect concentrations of GLP-1, GLP-2 or glycaemic control in this population^(19,20)^. Ethics approval for the study was gained from the Regional Ethics Committee for Medical and Health Research and registered at clinicaltrials.gov (No. NCT02569684). Those eligible and willing to take part signed a consent form. The study was performed in accordance with the ethical standards laid down in the 1964 Declaration of Helsinki and later amendments.

Patients were invited to participate through social media, the Diabetes Outpatient Clinic at Oslo University Hospital, posters in the hospital lobby and pharmacies, and were recruited from general practices. Eligibility for participation was determined at a screening visit at least 4 weeks in advance of enrolment. The subjects were adult men and women with type 2 diabetes and a BMI of ≤40 kg/m^2^. They were not treated with insulin or GLP-1 analogues and had an HbA_1c_ of <10⋅0% (86 mmol/mol). Exclusion criteria were weight changes of >3 kg within the last month, the performance of high-intensity exercise, pregnancy, fibre intake of >30 g/d, treatment with antibiotics within the last 3 months, drug or alcohol dependency, or the use of prebiotic or probiotic supplements. Subjects diagnosed with chronic diseases that may affect the outcomes or the subjects’ ability to participate were also excluded. The patients’ fibre intake was screened with a simplified approach where we asked how often they consumed specific food items known as important fibre sources in the Norwegian diet and their portion sizes.

**Fig. 1.**
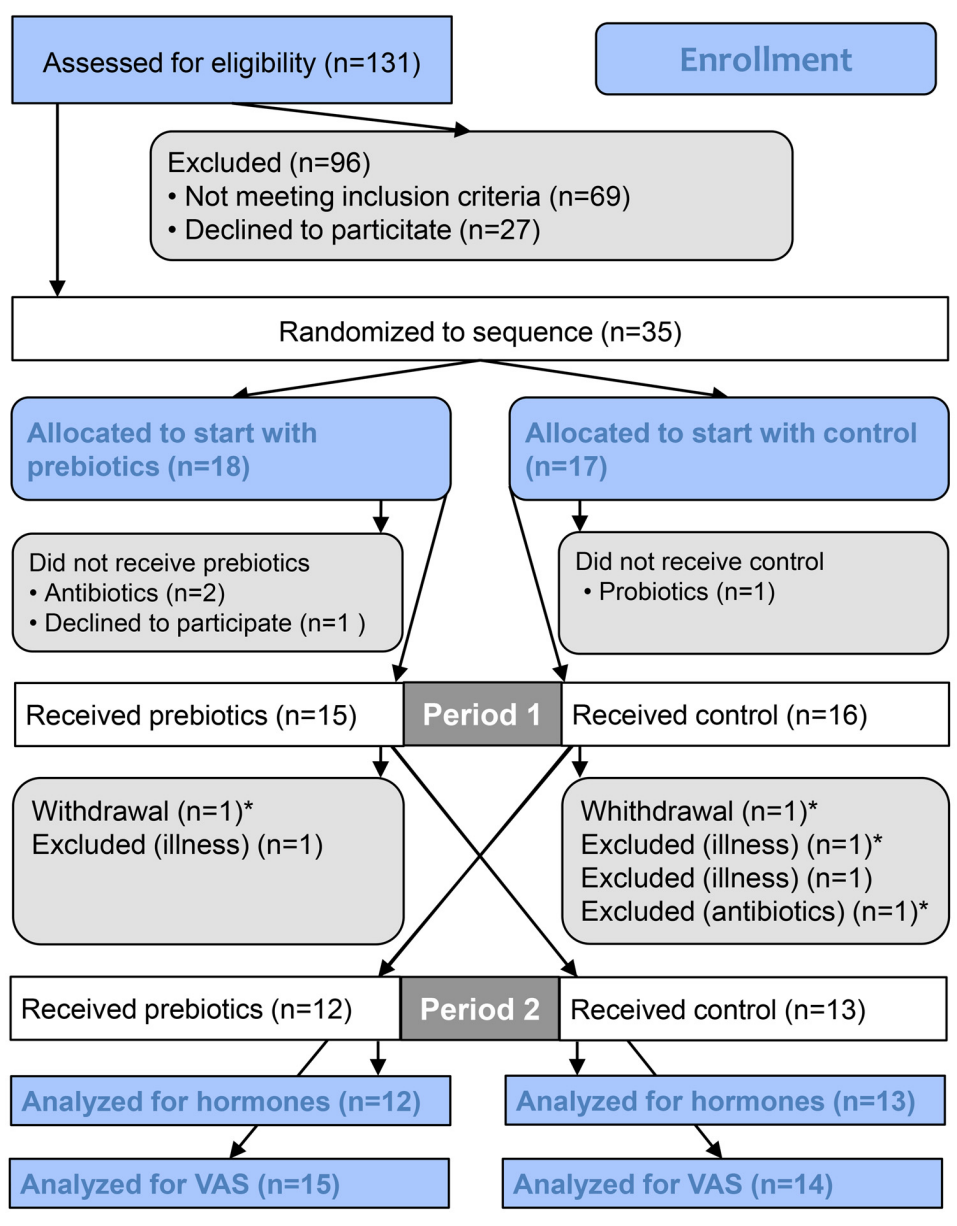
Flow chart showing all subjects approached for the study. *Included in analyses for appetite scores and portions. VAS, visual analogue scale.

We screened 131 patients for eligibility and 35 patients were randomised to start with either prebiotics or a control supplement (Fig. 1). Long distance from home was the main reason for not meeting the inclusion criteria. A total of twenty-nine participants were included in the analysis for the subjective ratings of appetite and energy intake. Blood analysis of hormones was performed only for the twenty-five participants that attended all four visits.

During the study, the participants were instructed to maintain their habitual lifestyles. Two days in advance of each visit, they were to stop taking diabetes medication. They were also told to avoid strenuous exercise 1 d in advance of the visits and to initiate fasting at midnight on the last evening before the visits.

### Dietary intervention

**Fig. 2.**
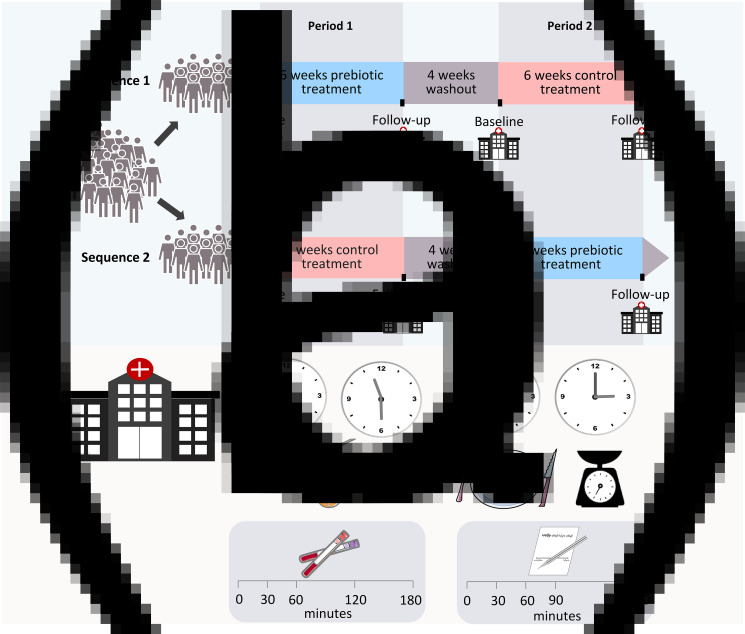
Overview of study design (a). Overview of time line for the standardised mixed meal and the *ad libitum* lunch during visits (b).

The participants consumed a daily supplement of 16 g ITF (a 50/50 mixture oligofructose and inulin; Orafti® Synergy1, Beneo GmbH, Germany, constituting 24 kcal) and a control supplement (16 g maltodextrin, AGENAMALT 20.222 Maltodextrin DE19, Agrana Stärke, Austria, constituting 64 kcal) in randomised order, in addition to their ordinary diet (Fig. 2(a)). A 4-week washout was included between the two intervention periods that lasted 6 weeks each. The powdered supplements were provided in identical, unlabelled and non-transparent sachets of 8 g, and were indistinguishable regarding appearance and taste. The participants consumed only one sachet per day for the first week to allow for adaptation, subsequently advancing to two sachets per day for the remaining 5 weeks. They were instructed to add the supplements to food or drinks and ingest whenever convenient. Unused sachets were returned, providing a measure of compliance.

### Data collection

Before and after the 6-week intervention periods, the participants met at the hospital for examinations after an overnight fast. Anthropometrics were assessed on arrival while still in a fasting state. Appetite hormones were assessed during the morning with a standardised mixed meal (Fig. 2(b)). Blood for the analysis of appetite hormones was collected in EDTA tubes in fasting and 30, 60, 120 and 180 min after consumption of two nutritional drinks (200 ml Fresubin 2 kcal Drink vanilla and 100 ml Fresubin Jucy Drink apple), consisting of 550 kcal, 78⋅5 g carbohydrate, 24 g protein and 15⋅6 g fat. The drinks were consumed within 12 min.

After the assessment of hormones, the participants were served an *ad libitum* lunch (Fig. 2(b)). The meal was a mixed casserole dish of pasta with meatballs and vegetables (Fjordland, ready meals), consisting of 114 kcal, 17⋅3 g carbohydrate, 4⋅9 g protein and 2⋅5 g fat/100 g, and pre-tested for palatability by hospital staff. The lunch was consumed within 30 min, and participants were instructed to eat as much as desired. They were allowed unrestricted amounts of water to accompany the meal. All food ingested was weighed and registered by the participants on a kitchen scale with 1⋅0 g accuracy. The subjective feeling of appetite was assessed before (time 0) and 30, 60, 90 and 180 min after meal initiation using the visual analogue scale (VAS)^(21–23)^. The participants answered four questions regarding hunger, satiety, fullness and prospective food consumption by drawing a vertical mark on a 100 mm line with opposing terms at the ends (Supplementary Table S1).

At the first baseline visit and both follow-up visits, the participants also delivered the food frequency questionnaire (FFQ) for the assessment of habitual diet.

#### Appetite hormones analyses

Forty μl DPP-IV inhibitors (Merck Millipore, Germany) and 40 μl Pefabloc® SC (Merck Millipore, Germany) were added to the EDTA tubes in advance. Plasma was separated by centrifugation at 3500 rpm at 4 °C for 10 min and aliquots stored at −80 °C in biobank for later analysis of acylated ghrelin and total peptide YY (PYY) at the Hormone Laboratory, Oslo University Hospital, Norway. Acylated ghrelin and total PYY were analysed in duplicates using Human Metabolic Hormone Magnetic Bead Panel (Metabolism Multiplex Assay, Merck Millipore, Germany) and Luminex 200 Technology (Invitrogen, Thermo Fisher, USA). The minimum detectable concentration of the assay was 13⋅7 pg/ml for acylated ghrelin and 41⋅2 pg/ml for PYY. For both hormones, the intra- and inter-assay coefficients of variation were <10 and <15%, respectively.

#### Anthropometrics

Height was measured with a standard stadiometer. Before and after the treatment periods, a body composition analyser (Tanita BC-418 MA Segmental Body Composition Analyser) was used for measuring weight and body impedance. Subjects were measured bare feet, wearing indoor clothing.

#### Assessment of habitual diet

Dietary data were obtained with a validated, self-administered, paper-based optical mark readable FFQ assessing total diet^(24,25)^. The participants were instructed to fill in questionnaires based on eating habits during the last 6 weeks.

#### Gastrointestinal symptoms

After finishing each treatment, the participants reported changes in gastrointestinal symptoms during the last 6 weeks by filling out a simple questionnaire where abdominal discomfort, constipation, diarrhoea, bloating and flatulence were rated as much worse, worse, unchanged, better or much better.

### Sample size

Since this was a substudy of a study investigating the effect of prebiotics on GLP-1 and glycaemic regulation, the power calculation was related to that outcome. With few data being available in the literature for a power calculation at the time the study was planned, the sample size was calculated based on changes in AUCs for GLP-1 response in patients with type 2 diabetes after a pharmaceutical intervention. The mean (95% CI) difference between treatment and placebo in the trial was 2⋅34 (1⋅32, 3⋅35) pmol/L × min^(26)^. This provided a tentative sample size of twenty-three individuals to achieve 80% power at *α* of 0⋅05. To account for drop-outs and a possible lower treatment effect due to differences in intervention and design, we added twelve persons, giving a total of thirty-five participants required for randomisation.

### Randomisation and blinding

Randomisation lists were generated by a statistician who had no further involvement in the trial, by using a randomisation command for two by two crossover studies in Stata version 14 software. Research personnel not directly involved in the study, administered the randomisation of subjects and product distribution. Treatment allocation was concealed for both participants and clinical investigators.

### Statistical analyses

SPSS version 26.0 software was used for descriptive statistics and analyses of biochemical responses, subjective ratings, portion sizes and adverse effects. Descriptive statistics are reported as mean (sd) or *n* (%) and results as estimated mean ± sem or with 95% confidence intervals, unless otherwise stated. Reported *P*-values are two-sided and *P* < 0⋅05 was considered significant for all tests.

Outcomes for the VAS and both hormones at the visits were determined by a linear mixed model for repeated measures using all available data at each time point. We analysed the mean differences in response between prebiotics and the control supplement (between treatments) and between baseline and 6 weeks (within treatments). We accounted for repeated measures according to best model fit. Fixed effects in the models were treatment (prebiotics/control), day (baseline/6 weeks), with their interactions, and minutes. The effects of period and carry-over (treatment-by-period interaction) were tested in all models and removed if not significant. As potential confounding factors, we investigated the effects of age, gender, baseline BMI and metformin. We also investigated the impact of portion size on VAS scores.

Total AUC for the VAS and hormones were calculated by the trapezoidal rule. Energy intake at the *ad libitum* lunch and the AUCs were analysed with the same approach as described earlier, but without minutes as a fixed effect.

Normality of residuals was evaluated with quantile–quantile plots and the Shapiro–Wilk test, and the outcome measures were transformed if appropriate.

## Results

### Descriptive

The mean (sd) age of the participants was 61⋅5 (11⋅7) years, BMI 28⋅9 ( 4⋅5) kg/m^2^ and diabetes duration 5⋅1 (4⋅4) years. At baseline, the fibre intake was 31⋅5 (10⋅2) g/d, and the mean HbA1c was 6⋅9 (1⋅0)% (52 mmol/mol) (Table 1). The baseline characteristics did not significantly differ between the twenty-five subjects that were included in the hormone analyses and the total study population.

**Table 1.** Subject characteristics at baseline^a^

Variable	*n* 25^b^		*n* 29^c^	
Men	15	(60⋅0)	17)	(58⋅6)
Age (years)	63⋅1	11⋅5	61⋅5	11⋅7
Fasting glucose (mmol/l)	8⋅7	2⋅4	8⋅8	2⋅4
BMI (kg/m^2^)	29⋅1	4⋅7	28⋅9	4⋅5
HbA_1C_ (mmol/mol)	6⋅9	1⋅0	6⋅9	1⋅0
HbA1C (%)	52		52	
Dietary fibre (g/d)	32⋅2	10⋅3	31⋅5	10⋅2
Diastolic blood pressure (mmHg)	137⋅8	18⋅2	136⋅3	17⋅9
Systolic blood pressure (mmHg)	85⋅7	10⋅1	85⋅6	9⋅5
Diabetes duration (years)	4⋅7	4⋅4	5⋅1	4⋅4
Diabetes treatment				
Diet	8	(32⋅0)	8	(27⋅6)
Metformin	17	(68⋅0)	21	(72⋅4)
SLGT2 inhibitors	2	(8⋅0)	4	(13⋅8)
DPP-4 inhibitors	5	(20⋅0)	7	(24⋅1)
Sulfonylureas	1	(4⋅0)	1	(3⋅4)

a Data are mean (sd) or *n* (%).

b Analysed for hormones.

c Analysed for appetite scores and portion sizes.

### Appetite hormones

#### Acylated ghrelin

**Fig. 3.**
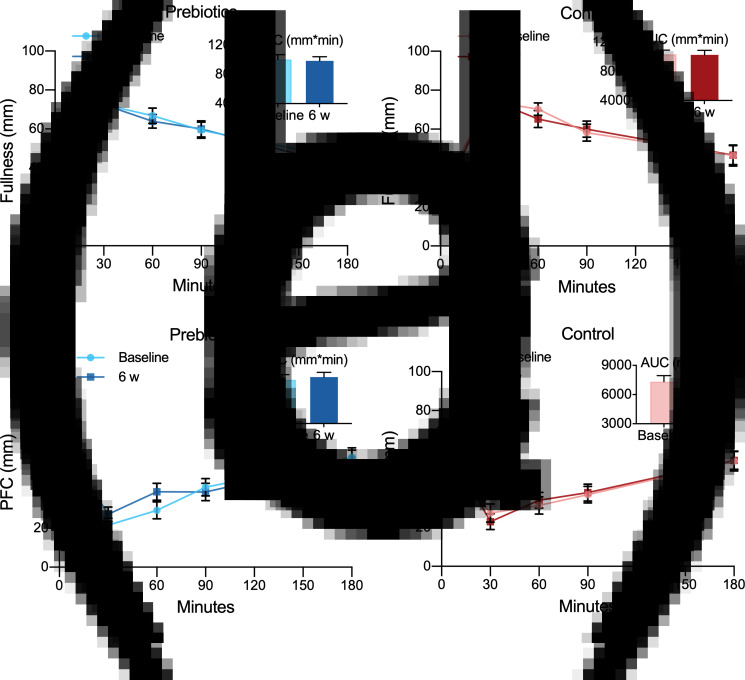
Plasma concentrations of acylated ghrelin (a, b) and total PYY (c, d) in response to a standardised mixed meal before (baseline) and after (6 weeks) treatment with prebiotics (a, c) and a control supplement (b, d). Values are predicted as means and sem. Insets are corresponding AUC values.

Responses to the test meal of acylated ghrelin in plasma did not change during treatment with prebiotic fibres or control (Fig. 3(a), (b); Tables 2 and 3).

**Table 2.** Effect of prebiotics and control supplement on hormones and appetite scores^a^

	Prebiotics				Control supplement				Time by treatment, *P*
	Baseline	95% CI	6 weeks	95% CI	Baseline	95% CI	6 weeks	95% CI	
Ghrelin^b^ (pg/ml)	37⋅42	35⋅51−39⋅33	39⋅14	37⋅15−41⋅14	40⋅51	38⋅44−42⋅57	34⋅86	33⋅08–36⋅64	0⋅089
PYY (pg/ml)	153⋅60	126⋅45−180⋅75	153⋅27	126⋅64−179⋅90	150⋅87	123⋅72−178⋅02	161⋅97*	135⋅34–188⋅60	0⋅013^†^
Hunger (mm)	32⋅72	27⋅74−37⋅69	35⋅59	30⋅71−40⋅46	31⋅62	26⋅66−36⋅58	36⋅67	31⋅78–41⋅56	0⋅366
Satiety (mm)	58⋅67	53⋅96−63⋅37	57⋅94	53⋅36−62⋅53	61⋅38	56⋅57−66⋅19	59⋅64	55⋅02–64⋅25	0⋅644
Fullness (mm)	55⋅30	49⋅16−61⋅44	54⋅15	48⋅47−59⋅82	58⋅27	52⋅02−64⋅53	56⋅09	50⋅37–61⋅80	0⋅683
Prospective food consumption (mm)	42⋅51	36⋅86−48⋅17	44⋅74	39⋅47−50⋅01	42⋅14	36⋅50−47⋅78	43⋅52	38⋅21–48⋅83	0⋅791

a Data are marginal means and 95% confidence intervals.

b Data analysis performed on natural log-transformed values. Back-transformed values are presented as geometric means and 95 % confidence intervals. *P* < 0⋅05.

* Significant effect within treatment: *P* = 0⋅013.

† Significant effect between treatments: *P* = 0⋅013.

**Table 3.** Effect of prebiotics and control supplement on hormones and appetite scores, AUCs^a^

	Prebiotics				Control supplement				Time by treatment, *P*
	Baseline	95% CI	6 weeks	95% CI	Baseline	95% CI	6 weeks	95% CI	
Ghrelin^b^ (pg/ml × min)	8⋅84	8⋅46−9⋅22	8⋅93	8⋅56−9⋅30	8⋅82	8⋅44−9⋅20	8⋅85	8⋅48−9⋅21	0⋅713
PYY (pg/ml × min)	27 903	23 357−32 449	27 632	22 979−32 285	27 752	23 108−32 296	29 780	25 131−34 429	0⋅130
Hunger (mm × min)	5510	4509−6512	6067	5047−7088	5462	4467−6458	6113	5285−7340	0⋅615
Satiety (mm × min)	10 746	9796−11 696	10 876	9934−11 818	11 278	10 302−12 254	11 097	10 148−12 046	0⋅559
Fullness (mm × min)	10 431	9205−11 657	10 022	8928−11 115	10 650	9395−11 905	10 493	9387−11 600	0⋅725
Prospective food consumption (mm × min)	7513	6329−8696	7916	6873−8960	7312	6133−8491	7697	6651−8743	0⋅981

a Data are marginal means and 95% confidence intervals.

b Natural log-transformed. *P* < 0⋅05.

#### Total PYY

The two treatments had different impacts on plasma concentrations of total PYY during the standardised meal (*P* = 0⋅013) (Fig. 3(c), (d); Tables 2 and 3). The PYY response increased by mean (sem) 11⋅1 (4⋅3) pg/ml (*P* = 0⋅01) after control treatment but remained unchanged after the prebiotic treatment. Throughout the trial, male participants had 58⋅7 (25⋅5) pg/ml higher concentrations of PYY than the females (*P* = 0⋅03), but of no consequence to the outcome (data not shown).

### Subjective rating of appetite

#### Hunger

**Fig. 4.**
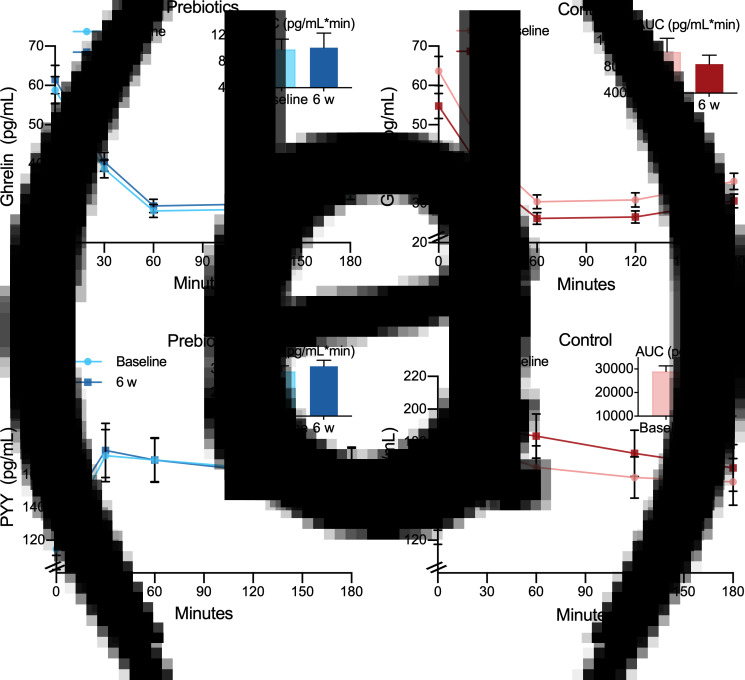
Appetite ratings of hunger (a, b) and satiety (c, d) assessed by the visual analogue scale in response to an *ad libitum* lunch before (baseline) and after (6 weeks) treatment with prebiotics (a, c) and a control supplement (b, d). Values are predicted as means and sem. Insets are corresponding AUC values.

The two treatments did not differ in effect on the subjective feeling of hunger (Fig. 4(a), (b); Tables 2 and 3).

#### Satiety

The two treatments showed no difference in effect on the satiety (Fig. 4(c), (d); Tables 2 and 3). The male participants rated satiety (mean ± sem) 21⋅4 (7⋅4) mm higher than the females throughout the trial (*P* = 0⋅001), but this did not change the outcome.

#### Fullness

**Fig. 5.**
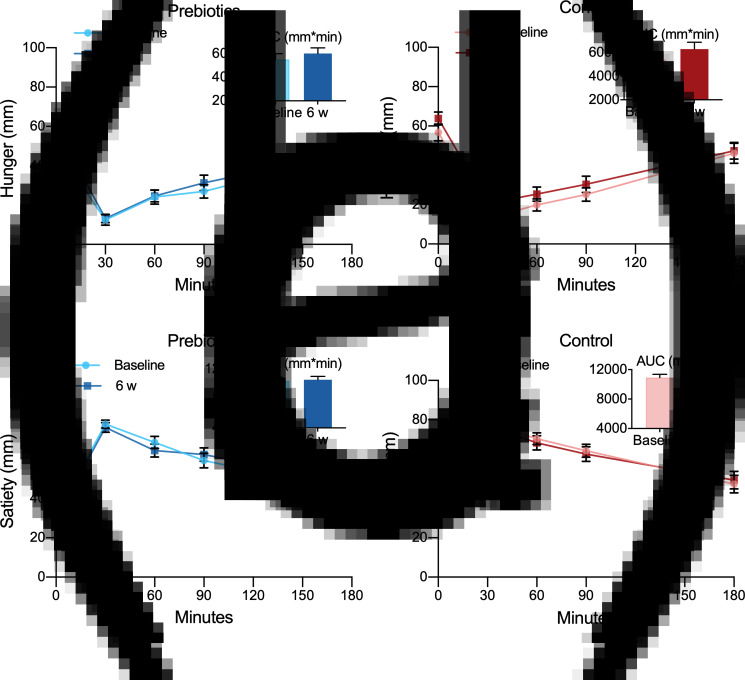
Appetite ratings of fullness (a, b) and prospective food consumption (c, d) assessed by the visual analogue scale in response to an *ad libitum* lunch before (baseline) and after (6 weeks) treatment with prebiotics (a, c) and a control supplement (b, d). Values are predicted as means and sem. Insets are corresponding AUC values. PFC, prospective food consumption.

The impact of the two treatments did not differ on fullness (Fig. 5(a), (b); Tables 2 and 3).

#### Prospective food consumption

There were no differences between the two treatments in effect on prospective food consumption (Fig. 5(c), (d); Tables 2 and 3).

### Energy intake and weight

Portion sizes were remarkably similar at each of the four visits with energy intake ranging between mean (sem) 498 (47) and 532 (46) kcal and no significant difference (*P* = 0⋅40). As expected, the male participants consumed more than the females from the *ad libitum* lunch throughout the trial with a mean difference of 200⋅6 (85⋅2) kcal (*P* = 0⋅03), although this did not change the outcome. The FFQ showed no changes in habitual energy intake during the trial and changes in body weight did not differ between treatments either (*P* = 0⋅865) with mean (95% CI) body weight changes of −0⋅17 g (−0⋅61, 0⋅26) after prebiotics and −0⋅12 kg (−0⋅56, 0⋅32) after the control supplement.

We found no differences between the baseline values before and after washout for any of the outcomes and no difference between treatments in effect on hormones or VAS ratings at individual time points (data not shown). The covariates of age, baseline BMI and metformin did not affect any of the models.

### Compliance

The compliance was excellent, and only mean values (range) of 3⋅3% (0–20⋅8) of the prebiotic sachets and 4⋅3% (0−22⋅1) of the control sachets were returned.

### Gastrointestinal symptoms

After the prebiotic treatment, sixteen participants (64 %) reported passage of gas and flatulence being worse or much worse than before, while only two of the participants (4 %) expressed the same complaints after treatment with the control supplement (*P* < 0⋅001). There were no significant changes in other adverse effects during the trial.

### *Post hoc* evaluation of sample size

We performed a *post hoc* evaluation of the sample size capacity to detect changes in appetite hormones, subjective ratings and energy intakes (accounted for correlating observations) using G*Power^(27)^. With a sample size of twenty-five participants in the hormone analyses, we had 80% power to detect a mean difference with the effect size of 0⋅59 at *α* of 0⋅05. With data from twenty-nine participants in the VAS analyses, we had 80 % power to detect a mean difference with the effect size of 0⋅54 at *α* of 0⋅05. These effect sizes only slightly exceed Cohen's convention for a moderate effect (*d* 0⋅3–0⋅5).

## Discussion

The addition of 16 g of ITF to the ordinary diet of subjects with type 2 diabetes for 6 weeks did not induce any change in investigated appetite hormones. Neither did it improve subjective measurements of appetite or reduce energy intake. We observed a difference in response of PYY that increased after the control diet and remained unchanged after the prebiotic fibre supplement.

Effects of ITF on ghrelin, PYY and subjective feeling of appetite appear only to have been studied in non-diabetic populations, and the results reported are inconsistent. Only one study assessed the effect of ITF on energy intake in type 2 diabetes^(28)^. Our results on ghrelin are in line with the findings of Rebello *et al.*, who reported unchanged ghrelin in overweight individuals after 4 weeks of treatment with 4 g inulin/d^(29)^. Parnell *et al.*, on the other hand, reported diminished AUC for ghrelin in overweight participants after 12 weeks of treatment with 21 g oligofructose/d^(30)^. In a dose-escalation trial by Pedersen *et al.* where twelve healthy, normal-weight adults consumed 0, 15, 35 and 55 g oligofructose/d for 1 week^(31)^, they found a significant dose-dependent relationship between ITF and ghrelin and near the significant effect on ghrelin, although not with 15 g/d. However, it should be noted that this was a preliminary dose-escalation study without a placebo-control, and with a relatively small sample size. Another detail that warrants attention is that the participants increased their doses over an expanding time period. Hence, the participants’ microbiota were exposed to ITF for a considerably longer duration with every dose-escalation, and the results should be interpreted with caution. Apart from the results from this dose-escalating study, studies investigating the effect of doses of ITF in-between 4 and 21 g on ghrelin concentrations in humans are lacking. Nevertheless, we cannot exclude the possibility that a dose higher than 16 g/d used in the present study may be necessary to suppress ghrelin. Our chosen dose of 16 g/d was planned to be sufficient to induce the desired changes in the gut microbiota and GLP-1 response with minimal gastrointestinal discomfort^(32–34)^.

Trials evaluating the effect on PYY also seem to support that the prebiotic effect of ITF is dose-dependent. Rebello *et al.* found no effect on PYY after treatment with 4 g ITF/d for 4 weeks^(29)^, while Parnell *et al.* showed the increased response of PYY after 12 weeks consumption of 21 g ITF/d^(30)^, with overweight adults in both trials. Pedersen *et al.* reported increased responses of PYY after 1 week of treatment with 35–55 g, but not with 15 g ITF/d. However, the limitations to the trial that was pointed out earlier apply also to these findings. Verhoef *et al.* found increased PYY response after 16 g, but not 10 g ITF/d for 13 d, with healthy adults attending both trials^(35)^. Only one trial interrupts this pattern by failing to detect an impact of 1-week treatment with 20 g ITF/d in patients with gastroesophageal reflux disease, but this trial only included nine participants^(36)^. In contrast to the present study, Verhoef *et al.* showed that a dose of 16 g ITF/d was sufficient in normal-weight adults when administered over a comparably short time span of 13 d^(35)^. This suggests that treatment with 16 g ITF/d for as long as 6 weeks should have enhanced the response of PYY in the present trial, and indicates that ITF may have a different impact in type 2 diabetes than in non-diabetic populations. None of these studies found a PYY-increasing effect of the control supplement maltodextrin, as we did and this supports our belief that this finding is an artefact. The maltodextrin dose used in our study constituted a daily amount of carbohydrates comparable to less than a tablespoon of sucrose per day for 6 weeks. The last intake of either of the supplements may have been a maximum amount of 16 g and at a minimum of 9 h before a mixed meal test. We consider it unlikely that 16 g maltodextrin could acutely affect PYY response 9 h after ingestion.

Our results on the subjective rating of appetite regulation are in accordance with several studies that found no effect of ITF^(30,35,37,38)^, but in contrast to other studies reporting beneficial effects^(29,31,34,39,40–42)^. These differences may be due to variation in study design, amount of fibres, differences between ITF in the degree of polymerisation and populations, and do not allow firm and general conclusions to be made at present.

The energy intake remained unchanged during the present trial. This is in agreement with other studies measuring changes in energy intake in type 2 diabetes after treatment with ITF^(43,44)^ and galacto-oligosaccharides^(45)^, whereas Dehghan *et al.* reported reduced energy intake in women with type 2 diabetes after 8 weeks of consumption of 10 g ITF/d^(28)^. Two research teams reported reduced energy intake, in healthy adults with normal weight treated for 2 weeks with 16 g ITF/d and in healthy overweight adults treated with 21 g ITF/d for 12 weeks^(30,39)^. However, most studies like us found unchanged energy intake after treatment with ITF when compared to placebo, seemingly regardless of dose or length of intervention^(31,34,35,40–42,46)^. Systematic reviews and meta-analyses of prebiotic effects on appetite regulation, suppression of energy intake and weight in humans address the inconsistency between studies, but conclude on possible favourable effects as well^(11,12,14,15,47)^. Weight loss is among the effects reported after consumption of ITF^(30,48)^. We wished to avoid this effect because it could have confounded other outcome measures. In the planning of the trial, we consequently decided against the intervention period exceeding 6 weeks.

Previously, we reported an increase in bifidogenic species accompanied by an increase in short-chain fatty acids concentrations in faeces after treatment with ITF in patients with type 2 diabetes^(19)^. These changes were modest, however, and apparently not of any consequence for circulating levels of appetite hormones or the subjective feeling of appetite as shown in the present study. An aberrant microbial environment in the gut, as reported in type 2 diabetes, may also need the prebiotic intervention of longer duration for bacterial species with essential capabilities to thrive and establish a well-functioning microbial community serving the human host.

The strengths of the present study include the randomised double-blind design and we accounted for medication known to affect the gut microbiota^(49,50)^. The fast initiated at midnight prior to the visits, as well as refraining from taking any diabetes medication 2 d in advance, promoted the equality of baseline conditions. The 4-week washout also minimised the risk of carry-over effects and was confirmed by the statistical analyses showing no treatment-by-period interaction or difference between baseline values before and directly after washout. Furthermore, the level of compliance appeared high, and we had no drop-outs related to the intervention.

The present study was primarily not powered to investigate appetite responses and the present analyses must be considered exploratory. However, a *post hoc* evaluation of our sample size showed that the present study had 80 % power to detect changes that were only slightly above moderate effect size. The observed changes in hormones and VAS scores after the prebiotic treatment were, however, mostly negligible and thus of little practical interest. We also acknowledge that allowing the participants to drink unlimited amounts of water to the *ad libitum* lunch and throughout the remaining 180 min of the test, may have influenced the food intake. On the other hand, so could limitation or standardisation of water allowance have done and it is likely that the participants drank approximately the same amount at all four test meals. Another limitation to the study was failure to analyse appetite hormones in the blood sampled from four participants that did not attend all four visits. By the time we realised that data from these blood samples could have been included in the statistical analysis, the laboratory analyses were already completed and the multiplex kits discarded. Also, the intake of dietary fibre assessed with the FFQ at the first visit (baseline) was higher than expected, which may impair the generalisability of the results. Although a previous study reports more pronounced bifidogenic response with higher habitual fibre intake^(51)^, this was not reflected in the present study sample^(19)^. As reported in our previous publication, no significant correlation was found between reported fibre intake and changes in gut microbiota^(19)^.

## Conclusions

In conclusion, we observed no effect of ITF on ghrelin, PYY, subjective feeling of appetite or energy intake in patients with type 2 diabetes after 6 weeks of treatment. Our findings suggest that supplementation with ITF would not be effective in reducing appetite in type 2 diabetes.

## Supplementary material

For supplementary material accompanying this paper visit http://dx.doi.org/10.1017/jns.2021.70. click here to view supplementary material
