# Supplementary material for: Effect of inulin-type fructans on appetite in patients with type 2 diabetes: a randomised controlled crossover trial
Source: J Nutr Sci. 2021 Sep 10;10:e72. doi: 10.1017/jns.2021.70 (PMC8453458; doi:10.1017/jns.2021.70)
Supplement: Supplementary file 1 [file S2048679021000707sup001.pdf]

Supplemental material

Effect of inulin-type fructans on appetite in patients with type 2 diabetes: A randomized controlled crossover trial

Eline Birkeland[1,2], Sedegheh Gharagozlian[1], Kåre I. Birkeland[2,3], Oda K. S. Holm[4], Per M. Thorsby[5], Anne-Marie Aas[1,2]

[1]Section of Nutrition and Dietetics, Department of Clinical Service, Division of Medicine, Oslo University Hospital, Norway, [2]Institute of Clinical Medicine, Faculty of Medicine, University of Oslo, Norway [3]Department of Transplantation Medicine, Oslo University Hospital, [4]Department of Nutrition, Faculty of Medicine, University of Oslo, Norway, [5]Hormone Laboratory, Department of Medical Biochemistry, Oslo University Hospital, Norway

Corresponding author: Eline Birkeland, eline.birkeland@ous-hf.no

Supplemental table 1 Questions in the visual analogue scale

**Questions**		**Answer at 0 mm**	**Answer at 100 mm**
Hunger:	How hungry do you feel?	I am not hungry at all	I have never been more hungry
Satiety:	How satisfied do you feel?	I am completely empty	I cannot eat another bite
Fullness:	How full do you feel?	Not full at all	Totally full
Prospective food consumption:	How much do you think you can eat?	Nothing at all	A lot
